# Endoscopic transorbital resection of the temporal lobe: anatomic qualitative and quantitative study

**DOI:** 10.3389/fnana.2023.1282226

**Published:** 2023-09-25

**Authors:** Andrea De Rosa, Alejandra Mosteiro, Giulia Guizzardi, Pedro Roldán, Jorge Torales, Jessica Matas Fassi, Luigi Maria Cavallo, Domenico Solari, Alberto Prats-Galino, Alberto Di Somma, Joaquim Enseñat

**Affiliations:** ^1^Division of Neurosurgery, Department of Neurosciences, Reproductive and Odontostomatological Sciences, Università degli Studi di Napoli “Federico II”, Naples, Italy; ^2^Department of Neurosurgery, Hospital Clinic, Barcelona, Spain; ^3^Department of Ophthalmology, Hospital Clínic de Barcelona, Universidad de Barcelona, Barcelona, Spain; ^4^Laboratory of Surgical Neuroanatomy, Faculty of Medicine, Universitat de Barcelona, Barcelona, Spain; ^5^Institut d'Investigacions Biomèdiques August Pi i Sunyer (IDIBAPS), Barcelona, Spain

**Keywords:** neuroanatomy, skull base, endoscopy, transorbital approach, temporal lobectomy

## Abstract

**Objective:**

Starting from an anatomic study describing the possibility of reaching the temporal region through an endoscopic transorbital approach, many clinical reports have now demonstrated the applicability of this strategy when dealing with intra-axial lesions. The study aimed to provide both a qualitative anatomic description of the temporal region, as seen through a transorbital perspective, and a quantitative analysis of the amount of temporal lobe resection achievable via this route.

**Material and methods:**

A total of four cadaveric heads (eight sides) were dissected at the Laboratory of Surgical Neuroanatomy (LSNA) of the University of Barcelona, Spain. A stepwise description of the resection of the temporal lobe through a transorbital perspective is provided. Qualitative anatomical descriptions and quantitative analysis of the amount of the resection were evaluated by means of pre- and post-dissection CT and MRI scans, and three-dimensional reconstructions were made by means of BrainLab^®^Software.

**Results:**

The transorbital route gives easy access to the temporal region, without the need for extensive bone removal. The resection of the temporal lobe proceeded in a subpial fashion, mimicking what happens in a surgical scenario. According to our quantitative analysis, the mean volume removed was 51.26%, with the most superior and lateral portion of the temporal lobe being the most difficult to reach.

**Conclusion:**

This anatomic study provides qualitative and quantitative details about the resection of the temporal lobe via an endoscopic transorbital approach. Our results showed that the resection of more than half of the temporal lobe is possible through this surgical corridor. While the anterior, inferior, and mesial portions of the temporal lobe were easily accessible, the most superior and lateral segment was more difficult to reach and resect. Our study serves as an integration to the current anatomic knowledge and clinical practice knowledge highlighting and also as a starting point for further anatomic studies addressing more selected segments of the temporal lobe, i.e., the mesial temporal region.

## 1. Introduction

Endoscopic transorbital surgery has nowadays been applied for the management of several skull base lesions (Yoo et al., 2021). Many anatomic studies have demonstrated the efficacy of this route in reaching, in a minimally invasive fashion, the ventro-lateral aspect of the middle cranial fossa (Guizzardi et al., 2022). One of the first structures that is easily reached during the approach is the temporal dura, which sits right behind the ventral aspect of the greater sphenoid wing and can be opened to expose the temporal lobe. The proximity of this region of the brain could make this route a suitable option for the management of lesions located in the temporal lobe. As a matter of fact, several clinical reports have been published demonstrating the applicability of the transorbital approach for the management of intraparenchymal temporal lesions (Park et al., 2021). Nevertheless, anatomic studies providing a qualitative evaluation of the total area of exposure of the temporal lobe and quantitative measures of the amount of temporal lobe resection obtainable with this route are lacking. Backing upon these premises, the aim of our study is to add qualitative and quantitative data about the anatomic exposure and resection of the temporal lobe through a superior eyelid endoscopic transorbital approach.

## 2. Material and methods

Anatomic dissections were performed at the Laboratory of Surgical Neuroanatomy (University of Barcelona, Spain). In total, four specimens (eight orbits) were cleaned from any blood clots, fixed with Cambridge solution, and injected with red and blue latex to highlight arterial and venous systems, respectively. Before and after dissection, all specimens underwent both a multi-slice helical computed tomography (CT) scan (SIEMENS Somaton GoTop software version VA30A-SP03) with 0.5 mm thick axial spiral sections and a 0° gantry angle and an MRI study to obtain a 3D reconstruction of the temporal lobes. Endoscopic transorbital approaches were performed using a surgical microscope for the skin phase and a rigid endoscope of 4 mm in diameter and 18 cm in length, with 0° and 30° lenses (Karl Storz, Tuttlingen, Germany) for the rest of the procedure. The endoscope was connected to a light source through a fiber optic cable (300 W Xenon, Karl Storz) and to an HD camera (Endovision Telecam SL; Karl Storz). Data were uploaded to the Medtronic^®^ Workstation System to allow for navigation guidance and point registration during dissection. A superior eyelid endoscopic transorbital approach was then performed as previously described (Di Somma et al., 2022b). The quantitative analysis of the amount of temporal lobe removed was performed using the BrainLab^®^ neuronavigation workstation by fusing the post- and pre-dissection MRIs of each specimen using an automated software for volume calculation.

### 2.1. Endoscopic transorbital approach to the temporal lobe

After a linear incision along a superior eyelid wrinkle, the orbicularis muscle is identified and dissected along its fibers until the white plane is reached. This plane is followed laterally until the exposure of the periosteum of the lateral orbital rim. The periosteum is incised and dissection proceeds in a subperiosteal-subperiorbital dissection to expose the lateral wall of the orbit. The endoscope can be inserted into the surgical field and the main landmarks of the endo-orbital phase of the approach were identified, namely, the inferior and superior orbital fissures. Drilling of the lateral wall of the orbit begins between the two fissures until the fascia of the temporalis muscle is exposed. As the drilling of the lateral orbital walls continues, a “V-shaped” osseous wall appears at the center of the field, limited superiorly by the sphenoid ridge, inferiorly by the inferior orbital fissure, medially by the sagittal crest, and laterally by the temporalis muscle. The center of this “V” corresponds to the anterior portion of the middle cranial fossa. Gentle thinning of this area reveals the dura mater of the temporal pole, which must be detached from the floor of the middle cranial fossa. Once the dura of the temporal pole is exposed and detached from the surrounding bone, extensive flattening of the floor of the middle cranial fossa is a necessary step to improve the surgical freedom once the dura is opened, and the approach is carried out intradurally. The dura of the temporal pole is opened in a cruciate fashion, and tuck-up stitches are applied to keep it opened. At this point, a subpial resection of the temporal lobe can be started.

## 3. Results

### 3.1. Qualitative analysis

To make our results more clear and reproducible, we summarized our dissection in five sequential steps: steps 1 and 2 correspond to temporal pole exposure and identification of the four surfaces of the temporal lobe, respectively; step 3 is represented by the initial anterior corticectomy at the level of the temporal pole; step 4 begins with isolation and division of the main vascular feeders and the identification of the subpial plane in which the resection proceeds; the final step 5 corresponds to the identification of the posterior limits of the resection as seen from the transorbital perspective.

#### 3.1.1. Steps 1 and 2

**Figure 1:**
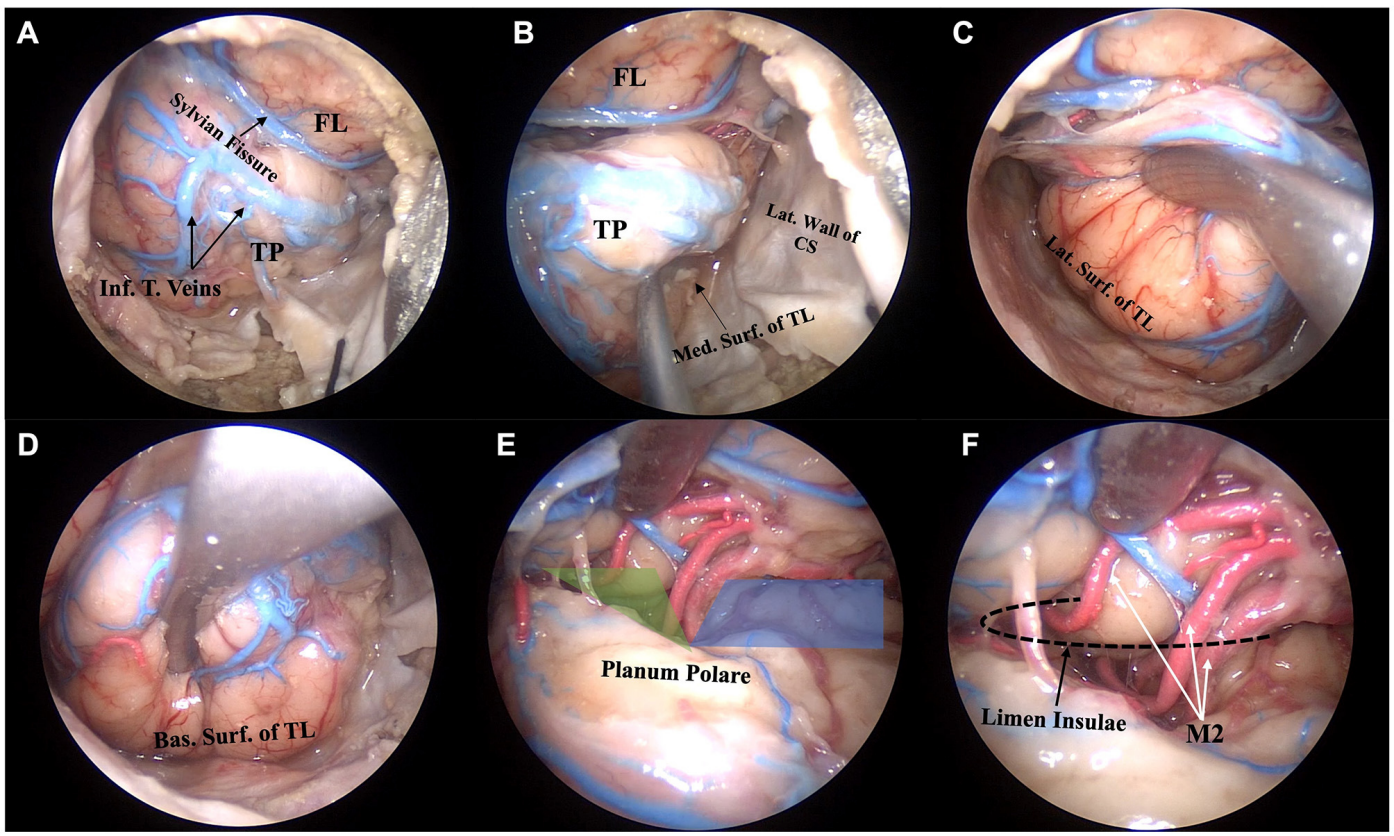
Anatomic pictures showing a right endoscopic transorbital approach for the exposure and resection of the temporal lobe. Step 1 and 2, corresponding to the exposure of the temporal pole and the four surfaces of the temporal lobe, are shown. After dural opening and application of tucking sutures, the temporal pole, with its vascular network, comes into view **(A)**. The mesial surface of the temporal lobe faces the lateral wall of the cavernous sinus inferiorly and the tentorial incisura superiorly **(B)**. The lateral surface of the temporal lobe is detached from the lateral wall of the middle cranial fossa **(C)**. After extradural flattening of the floor of the middle cranial fossa, the intradural exposure of the basal surface of the temporal lobe can be achieved **(D)**. The sylvian fissure is split to expose the superior surface of the temporal lobe, the sphenoid (shaded blue area) and operculoinsular (shaded green area) compartments of the sylvian cistern, and the branches of the middle cerebral artery **(E)**. The genu of the middle cerebral artery, with the M2 branches turning around the limen insulae, which sits at the lateral edge of the sphenoid compartment of the sylvian fissure, can be visualized **(F)**. Bas. Surf. of TL, basal surface of the temporal lobe; FL, frontal lobe; Inf. T. Veins, inferior temporal veins; Lat. Surf. Of TL, lateral surface of the temporal lobe; Lat. Wall of CS, lateral wall of the cavernous sinus; M2, insular segment of the middle cerebral artery; Med. Surf. Of TL, medial surface of the temporal lobe; TP, temporal pole.

After the dura opening, the temporal pole, along with its arterial and venous vascularization, comes into view. Before arachnoid dissection, the intradural exposure of the temporal lobe is limited to four surfaces: the temporal pole, the lateral surface, limited by the sphenoid and temporal bone, the basal surface, limited by the floor of the middle cranial fossa, and the medial surface, limited by the lateral wall of the cavernous sinus inferiorly and by the tentorial incisura superiorly. On the anterior surface of the temporal lobe, the inferior temporal and the temporo-sylvian veins can be displayed. After arachnoid dissection of the Sylvian fissure, the superior surface of the temporal lobe, with the *planum polare* and *temporale*, can be observed. In the depth of the Sylvian cistern, the path of the middle cerebral artery can be followed from the M1–M2 bifurcation to the origin of the M3 segments which wrap around the limen insulae to reach the surface of the insula (Figure 1).

#### 3.1.2. Steps 3 and 4

**Figure 2:**
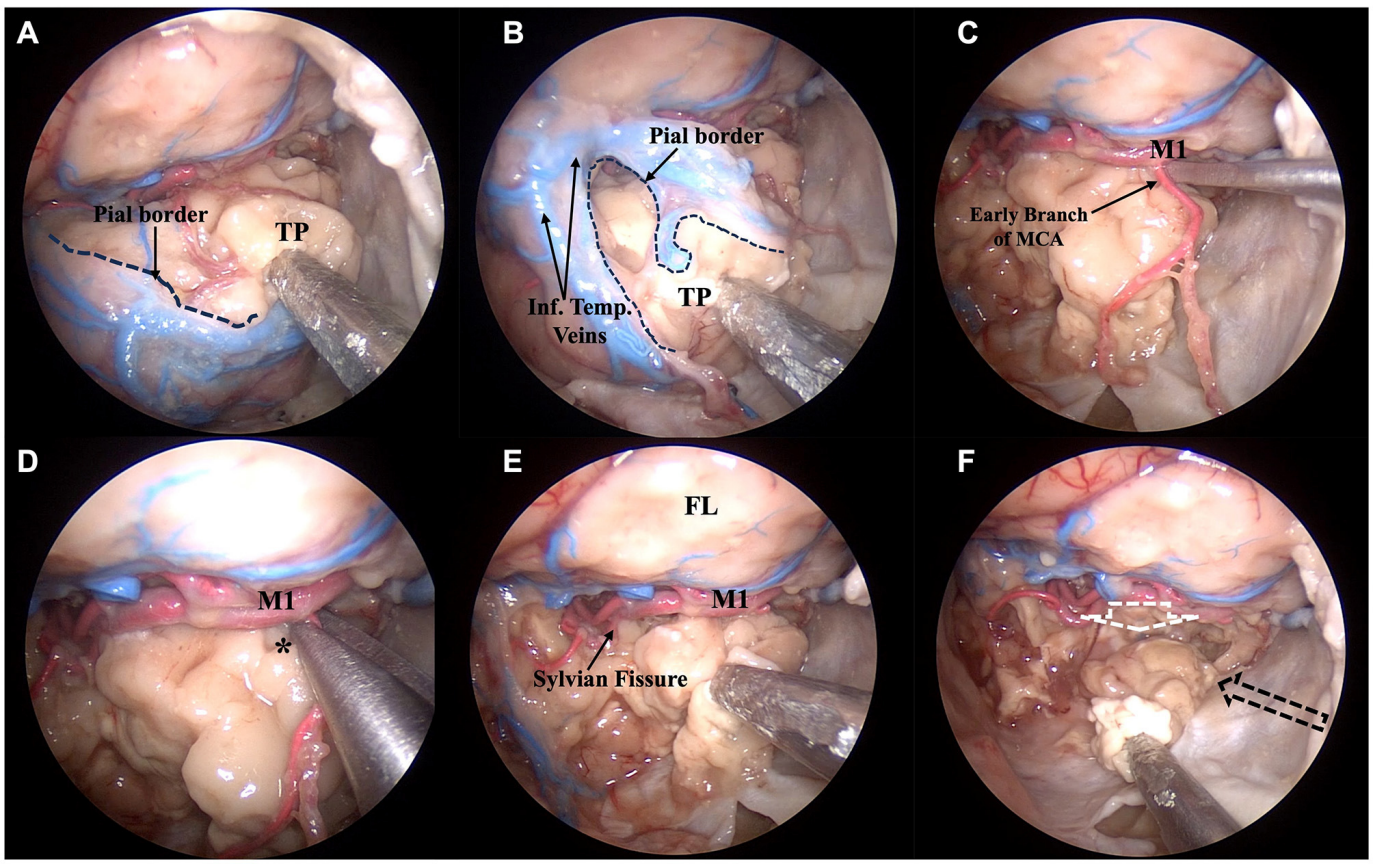
Anatomic pictures showing a right transorbital endoscopic approach for the resection of the temporal lobe. Steps 3 and 4, corresponding to the anterior corticectomy **(A, B)** and subpial dissection **(C–F)**, respectively, are demonstrated. After the identification of the temporal pole and identification of all the surfaces of the temporal lobe, a small incision over the pia mater covering the temporal pole is made **(A)** and resection of the temporal lobe thus proceeds in a subpial plane **(B)**. Mimicking what happens in a surgical scenario, the main vessels nourishing the temporal lobe are identified **(C)** and resected **(D**, *) before resection is continued **(E)**. The direction of the resection parallels the sylvian fissure superiorly (white dotted arrow), the lateral wall of the cavernous sinus and tentorial incisura, medially (black dotted arrow), and the middle fossa floor inferiorly (direction given by the surgical aspirator) **(F)**. * Early branch of the middle cerebral artery being dissected; FL, frontal lobe; Inf. Temp. Veins, inferior temporal veins; M1, sphenoid segment of the middle cerebral artery; TP, temporal pole.

To apply the same principles used during surgical procedures, the resection of the temporal lobe was made sequentially, trying to respect the principles of subpial dissection, and after resecting the main arterial branches which support its vascularization. Resection of the temporal lobe proceeded in an anterior-to-posterior direction and followed the plane parallel to the sylvian cistern superiorly and the tentorial incisura medially (Figure 2).

#### 3.1.3. Step 5

**Figure 3:**
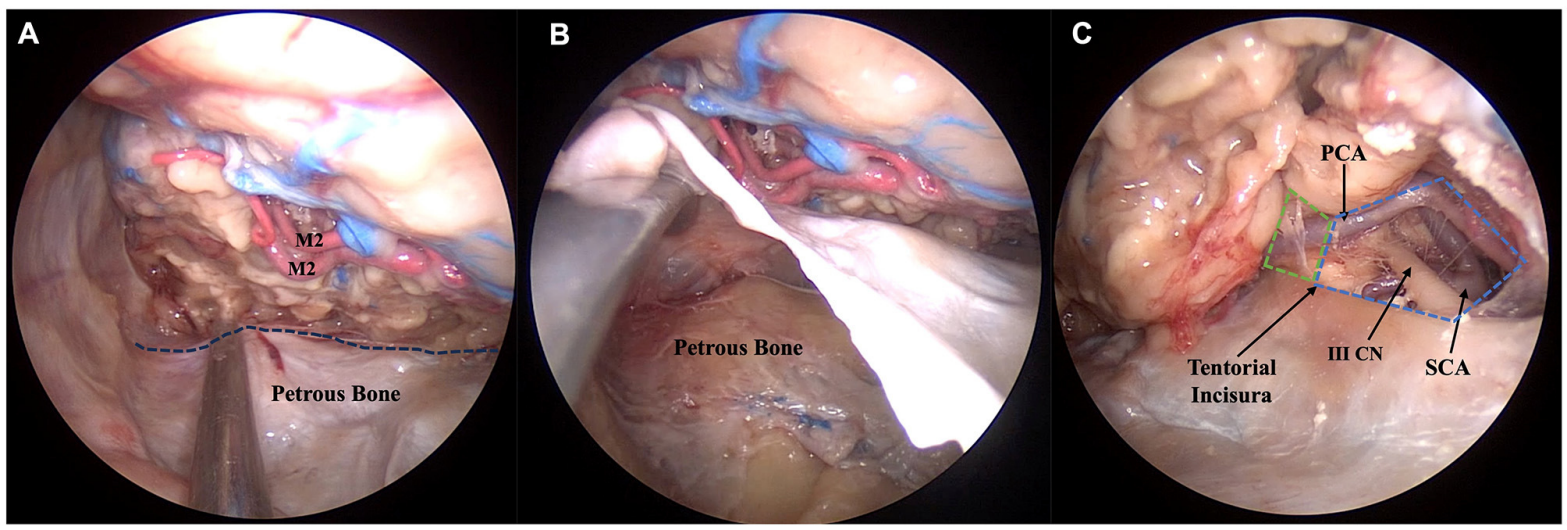
Anatomic pictures showing a right endoscopic transorbital approach for the resection of the temporal lobe. Step 5, corresponding to the final exposure of the posterior landmarks identified after maximal resection of the temporal lobe, is represented. As the posterior limits of the temporal lobe are poorly represented and difficult to identify, we selected as the postero-lateral limit of our resection the petrous bone, whose position can be verified intradurally with a surgical instrument pointing at the petrous ridge (dotted line) **(A)**. In **(B)**, with an extradural approach to the middle cranial fossa, the dura mater is elevated in order to confirm our position **(B)**. The postero-medial limit is represented by the junction between the crural (green dotted area) and interpeduncular cisterns (blue dotted area) **(C)**. III CN, oculomotor nerve; M2, insular segment of middle cerebral artery; PCA, posterior cerebral artery; SCA, superior cerebellar artery.

Temporal lobe removal was stopped right posterior to the intradural projection of the superior margin of the petrous bone. The right position of the petrous bone was also confirmed extradurally by detaching and elevating the dura of the middle cranial fossa. The most medial and posterior limit of the resection was represented by the interpeduncular and crural cisterns, whose contents were also exposed: the ponto-medullary junction, the posterior cerebral and superior cerebellar arteries, and the cisternal portion of the oculomotor nerve, which can be followed right to its entering into the oculomotor triangle on the roof of the cavernous sinus (Figure 3).

### 3.2. Quantitative analysis

**Figure 4:**
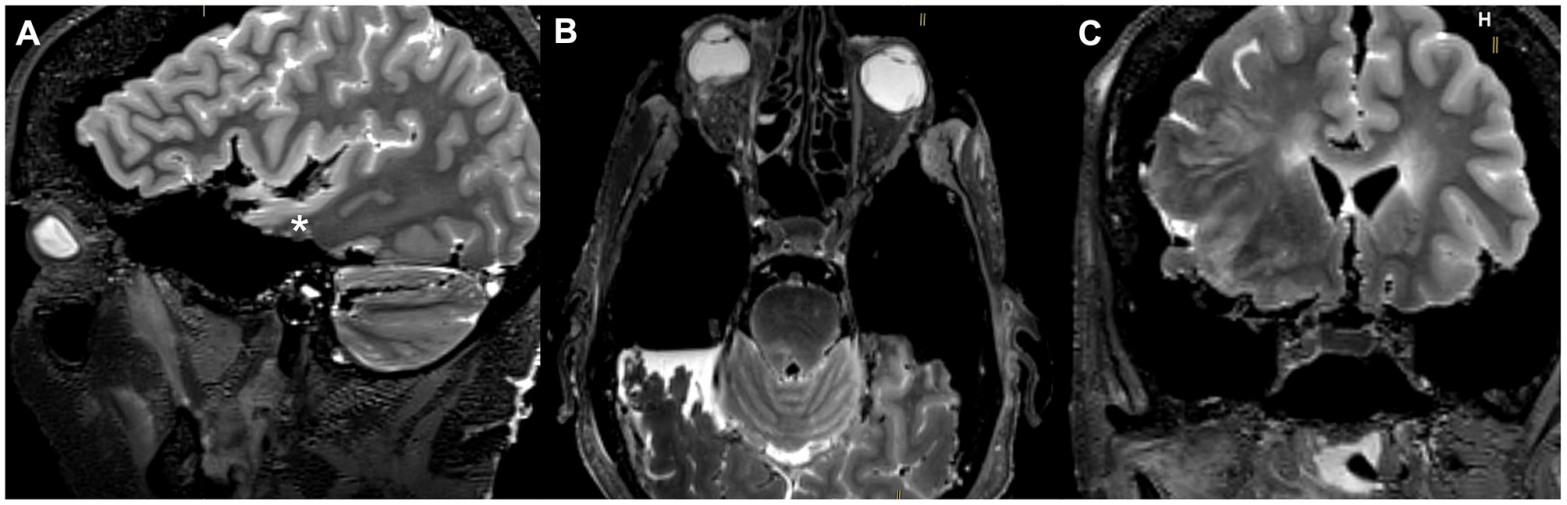
Post-dissection sagittal **(A)**, axial **(B)**, and coronal **(C)** T2-weighted MRI scans of one anatomic specimen showing the removal of the temporal lobe bilaterally, obtained after an endoscopic transorbital approach. The temporal pole and the mesial and inferior portions of the temporal lobe were fully resected. The resection of the superior temporal gyrus was limited to its anterior third, while its postero-lateral portion (*) represented the largest amount of temporal lobe left intact.

**Figure 5:**
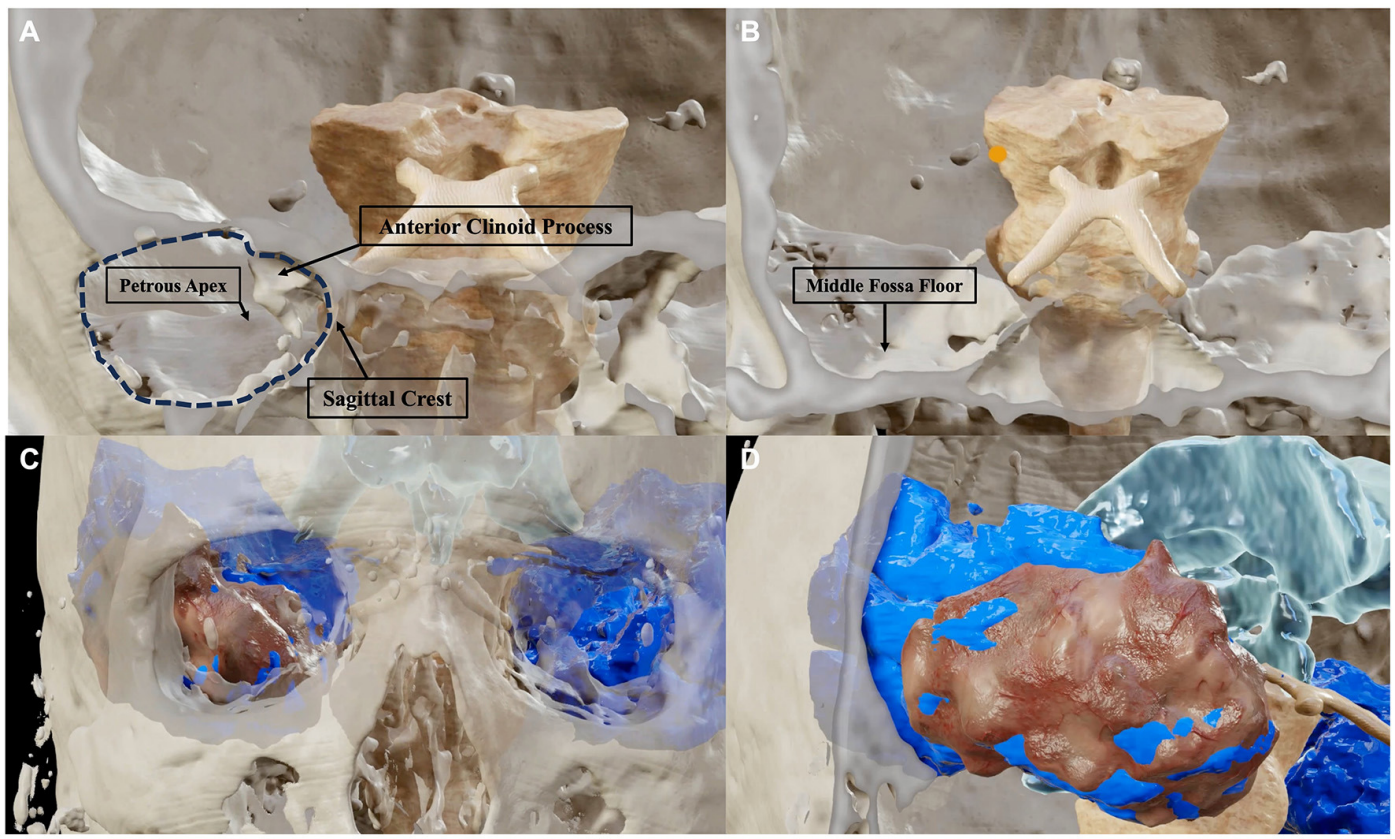
BrainLab^®^ reconstructions of post-dissection CT and MRI scans highlighting the relationship between the amount of bone removal **(A, B)** with the amount of resection of the temporal lobe **(C, D)**. The bone work of the approach is limited to an anterior sphenoidal craniotomy (dark dotted area) which provides access to the temporal pole. Three of the four bony pillars of the approach, namely, the sagittal crest, the anterior clinoid process, and the petrous apex are left intact **(A)**. The middle cranial fossa floor, which represents the fourth bony pillar, must be flattened to provide adequate maneuverability for the instruments which must work in parallel to the basal surface of the temporal lobe **(B)**. The amount of removal of the temporal lobe (brown reconstruction) in relation with the total of the temporal lobe (blue reconstruction) is shown in an anterior **(C)** and antero-lateral **(D)** perspective.

**Figure 6:**
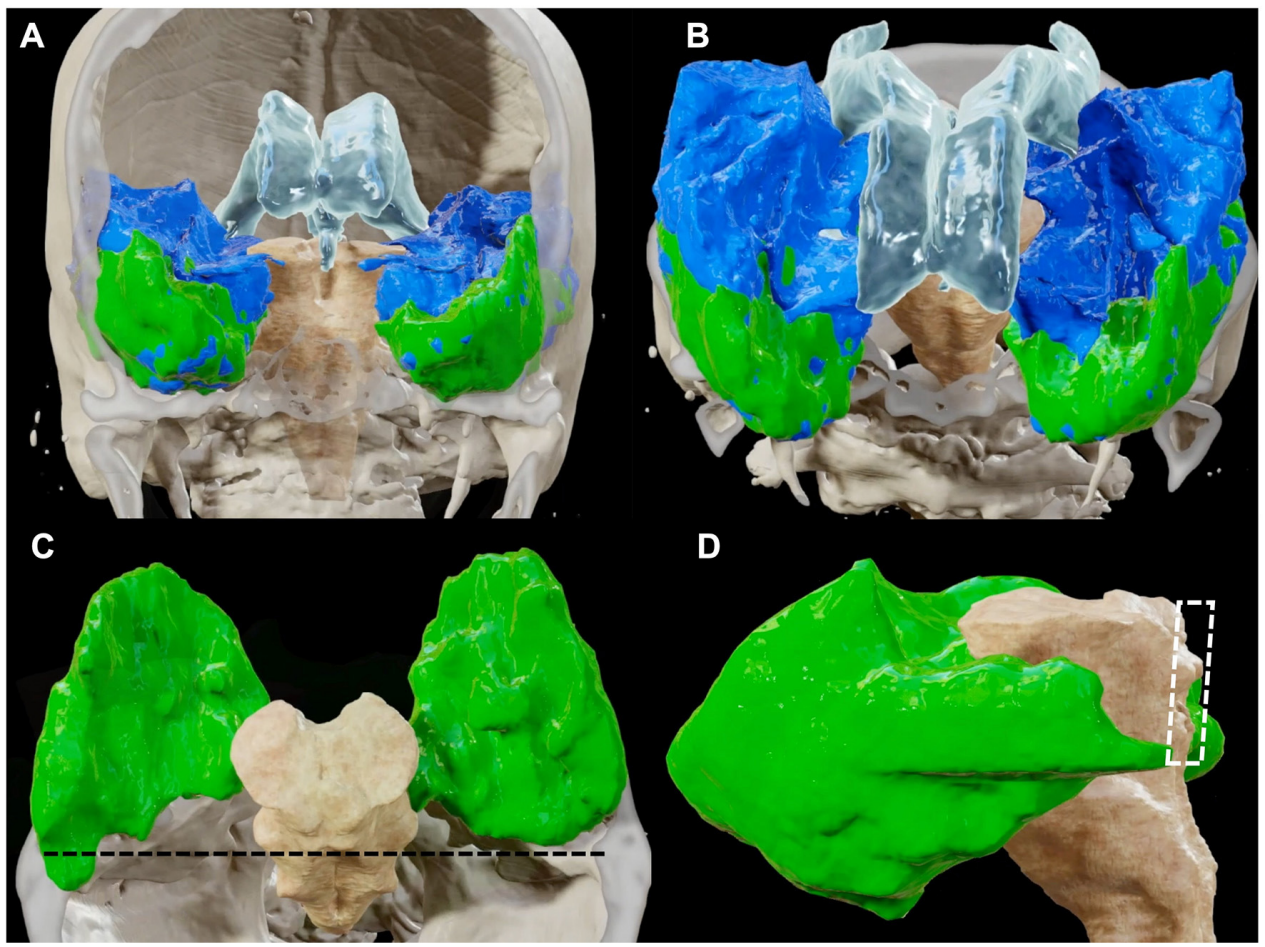
BrainLab^®^ reconstructions retrieved from post-dissection MRI quantifying the amount of temporal lobe removed (in green) in relation with the intact temporal lobe (in blue), seen from an anterior **(A)** and superior **(B)** perspective. In **(C)** and **(D)**, only the temporal lobe removed was left to highlight the relationships of the posterior limits of the resection with the surrounding brain structures. The posterior limit of the resection is represented by a plane tangential to the lamina quadrigemina [dark dotted line in **(C)** and white dotted area in **(D)]** as it is demonstrated in a superior **(C)** and lateral **(D)** view. The qualitative analysis of our resection shows that while the middle and inferior temporal gyruses together with the basal and medial portion of the temporal lobe represented the largest amount of the resection, the posterior two-thirds of the superior temporal gyrus were left intact.

With minimum bone removal, limited to flattening of the middle cranial fossa floor and preserving the three bony pillars of the transorbital approach, namely, the sagittal crest, the anterior clinoid process, and the petrous apex, we observed that the most inferior and mesial portion of the temporal lobe was easily accessible for the resection, while the most superior and lateral portion, corresponding to the posterior part of the superior temporal gyrus, represented a limited area for the maneuverability of the instruments (Figures 4, 5). The volume of temporal lobe removal was calculated with BrainLab^®^ Neuronavigation Software, by comparing, for each side of each specimen, the volumes of temporal lobe removed, calculated on post-dissection MRIs with the volume of the temporal lobes calculated on pre-dissection MRIs, calculated by means of an automated software of BrainLab^®^ workstation which allowed to selectively isolate the temporal lobes. We obtained eight measures from four specimens (Table 1), and the mean removed volume was 51.26% (Figure 6).

**Table 1:** Quantitative analysis of temporal lobe removal in both sides of four specimens obtained during an endoscopic transorbital approach.

**Specimen**	**Side**	**Volume of the temporal lobe (cm^3^)**	**Volume of the temporal lobe removed (cm^3^)**	**% of removal**
1	R	71	40	56.30%
	L	64.2	30.6	47.66%
2	R	68.4	31.7	46.34%
	L	65.2	34.2	52.45%
3	R	72.6	41.4	57.02%
	L	71.2	36.7	51.54%
4	R	69.3	31.7	45.74%
	L	70.7	37.5	53.04%
	Mean	69.07	35.47	51.26%
	Standard deviation	2.98	4.05	

## 4. Discussion

The endoscopic transorbital approach has now entered the skull base neurosurgeon's armamentarium for the management of selected skull base lesions (Somma et al., 2021; Yoo et al., 2021; Han et al., 2023). Starting from anatomic studies that demonstrated the feasibility of exploring via the transorbital route, the anterior (Di Somma et al., 2018; Nannavecchia et al., 2021; Lim et al., 2022), middle (Chibbaro et al., 2021; Guizzardi et al., 2022; Corvino et al., 2023), and posterior cranial fossae (De Rosa et al., 2022), many applications of this approach in the management of extradural lesions have been published (Dallan et al., 2018b; Zoia et al., 2018; Park et al., 2020a; Corvino et al., 2022; Lee et al., 2022; Lim et al., 2022; Noiphithak et al., 2022; Di Somma et al., 2023a; Zoli et al., 2023), demonstrating the possibility of overcoming many concerns attributed to this approach in terms of poor maneuverability and intraoperative complications' management (Kim W. et al., 2021; Di Somma et al., 2023b).

Parallel to the development of this technique for the management of extra-axial lesions and considering the “easy-access pathway” given by the transorbital corridor to the temporal region, the possibility of managing intra-axial pathologies and performing resection of selective parenchymal has also been investigated (Chen et al., 2014, 2015; Dallan et al., 2018a; Kim E. H. et al., 2021; Câmara et al., 2023). In particular, Chen et al. demonstrated with an anatomic investigation the feasibility of the transorbital approach in dissecting the mesial temporal lobe structures in a subpial manner and pointed out the advantages of early visualization of the medial structures, i.e., oculomotor and trochlear nerves, and the avoidance of exposure of the temporal stem by the transorbital approach, theoretically reducing the risk of postoperative complications when comparing to the standard craniotomy approach (Chen et al., 2014). More recently, Camara et al. have also proposed a modification of the standard superior eyelid approach in resecting selectively the mesial temporal lobe through an infero-lateral pre-septal approach without violating the neocortex or the roof of the temporal horn (Câmara et al., 2023).

Anatomic results have been promptly applied in recent clinical series demonstrating the possibility of using the transorbital approach to address intra-axial pathologies of the mesial temporal lobe (Chen et al., 2015; Park et al., 2021). In the first case series of seven patients harboring temporal gliomas, Park et al. provided their surgical outcomes with the use of an endoscopic transorbital approach. Their experience shows that a total resection of the temporal lobe, when needed, is also possible by using this technique (Park et al., 2021). It is interesting to underline that surgery of the mesial temporal lobe for the management of drug-resistant epilepsy, which has always been addressed by means of standard craniotomy approaches (Falconer et al., 1955; Olivier, 2000; Adada, 2008; Thudium et al., 2010), is now considered a valuable indication for the use of minimally invasive approaches (Kalinin et al., 2017; Park et al., 2020b; Gardner et al., 2023; Gonzalez-Martinez et al., 2023), with the transorbital route being one of them (Phillips et al., 2023).

We are then moving through the application in a clinical scenario of the third stage of the levels of difficulty of the endoscopic transorbital approach, as described by Di Somma et al. (2022a), which deals with the intra-axial lesions located in the temporal lobe. Nevertheless, even if qualitative anatomic studies have been published depicting the intradural anatomy of the temporal region through a transorbital perspective, quantitative data and technical nuances of a “tailored” transorbital approach for intra-axial lesions are still lacking. In this context, our manuscript aims to provide such quantitative integration to the current knowledge, stimulating in this way its understanding and application. Concerning the technical aspects of the procedure, it must be considered that even if the endoscopic transorbital approach is primarily applied for skull base lesions, the amount of “bone work” must be tailored to balance the need for maneuverability and the need for bone removal to reach the target, represented by the temporal lobe in this case. The “bone work” of the transorbital approach relies on the need to remove the four bony pillars of the approach, represented by the sagittal crest, the anterior clinoid process, the middle fossa floor, and the petrous apex. When dealing with pathologies of the parasellar area or the anterior cranial fossa, the removal of the sagittal crest and anterior clinoid process, during a transorbital approach, represents a fundamental step to gain adequate surgical freedom for surgeons' maneuverability (Corrivetti et al., 2022; Lim et al., 2022). Recently, our group also demonstrated the possibility of reaching the postero-lateral aspect of the skull base and tentorial region through the transorbital pathway, and in this case, three of the bony pillars, namely, the sagittal crest, middle fossa floor, and petrous apex, must be partially or totally removed to gain adequate working space for instruments insertion and maneuverability (De Rosa et al., 2022). On the other hand, we observed that adequate surgical maneuverability and working space can be obtained when dealing with the intradural space.

In our anatomic dissection, the bone opening was limited to an oval-shaped craniectomy of the greater sphenoid wing, large enough to expose the dura of the temporal pole, and to an extensive flattening of the middle cranial fossa floor which allowed for better dura mobilization and an easier insertion of the instruments parallel to the inferior aspect of the temporal lobe. There was no need to completely remove the sagittal crest nor the drilling of the anterior clinoid process or the petrous apex. After dura opening, we achieved our dissection in a subpial fashion, mimicking what happens in a surgical scenario, following the boundaries of the temporal lobe, such as the cavernous sinus and tentorial incisura, medially, the Sylvian cistern superiorly, the middle fossa floor inferiorly, and the temporal bone, laterally. As the temporal lobe continues with the parietal and occipital lobe without a clear anatomic demarcation, the posterior limits of the temporal lobe are represented, conventionally, by the parieto-temporal line, on the lateral surface of the brain, and by the parieto-occipital sulcus on the medial surface. During endoscopic transorbital resection of the temporal lobe, it was not possible to clearly define a posterior limit of the resection without the use of neuronavigation. We decided to stop posteriorly our dissection at the level of the intradural projection of the petrous apex inferiorly and at the level of the lamina quadrigemina superiorly. The petrous ridge was identified by detaching the dura of the middle cranial fossa floor and checking its position extradurally, while the lamina quadrigemina was identified with the use of neuronavigation. At the end of the dissection, quantitative measurements showed a mean removal of 51.26% of the temporal lobe achievable by means of the transorbital approach. Particularly, the biggest amount of residual temporal lobe was the most superior and posterior portion. These results can be explained by the difficulty in defining the posterior limit of the dissection and by the increasing difficulty in instrument maneuverability at the deepest portion of the surgical field.

In the present study, we achieved the resection of the largest amount of the temporal lobe achievable with the minimum of bone work, but future studies could be planned to provide a stepwise resection of certain areas of the temporal lobe after sequential resection of the main “bony pillars” of the approach (i.e., the sagittal crest, middle fossa floor, anterior clinoid process, and petrous apex), thus providing a stratified “volumetric map” that could guide surgeons in identifying the sequence of steps needed to reach certain areas of the temporal lobe.

### 4.1. Study limitation

This study must be set among the series of anatomic highlights that are being published to keep shedding light on new insights into the endoscopic transorbital approach. As happens for all anatomic studies, the translation of results to a clinical setting is always limited to the critical differences that the laboratory setting provides with respect to the “real” surgery. The difference in brain tissue consistency and the lack of intraoperative bleeding are two of the main criticisms that hinder a direct application in surgery. Nevertheless, our manuscript aims to provide further quantitative information that can be useful in guiding future applications, considering the already available clinical data.

## 5. Conclusion

Due to its proximity to the temporal lobe, the endoscopic transorbital approach has been gaining more and more interest in the management of intra-axial lesions of the temporal region. Even though anatomic and clinical studies demonstrating its applicability are already available, our manuscript aims to provide a qualitative and quantitative analysis of the total resection of the temporal lobe via this route. Considering the increasing interest in minimally invasive strategies to manage pathology of the temporo-mesial region, i.e., drug-resistant epilepsy, selective anatomic studies comparing the different suitable approaches are necessary to underline the advantages and drawbacks of each strategy.

## Data availability statement

The raw data supporting the conclusions of this article will be made available by the authors, without undue reservation.

## Author contributions

ADe: Investigation, Writing—original draft, Data curation. AM: Investigation, Writing—original draft. GG: Investigation, Writing—original draft. PR: Supervision, Writing—review and editing. JT: Writing—review and editing, Conceptualization. JM: Supervision, Writing—review and editing. LC: Supervision, Validation, Writing—review and editing. DS: Writing—review and editing, Supervision, Validation. AP-G: Writing—review and editing, Formal Analysis, Resources. ADi: Supervision, Writing—review and editing, Conceptualization, Validation. JE: Funding acquisition, Supervision, Writing—review and editing.
